# Ethnicity-specific obesity cut-points in the development of Type 2 diabetes – a prospective study including three ethnic groups in the United Kingdom

**DOI:** 10.1111/dme.12576

**Published:** 2014-10-01

**Authors:** T Tillin, N Sattar, I F Godsland, A D Hughes, N Chaturvedi, N G Forouhi

**Affiliations:** 1 UCL Institute of Cardiovascular Science, University College LondonLondon; 2 Institute of Cardiovascular and Medical Sciences, University of Glasgow School of MedicineGlasgow; 3 Endocrinology and Medicine, Department of Medicine, Imperial College LondonLondon; 4 MRC Epidemiology Unit, University of Cambridge School of Clinical MedicineCambridge, UK

## Abstract

**Aims:**

Conventional definitions of obesity, e.g. body mass index (BMI) ≥ 30 kg/m^2^ or waist circumference cut-points of 102 cm (men) and 88 cm (women), may underestimate metabolic risk in non-Europeans. We prospectively identified equivalent ethnicity-specific obesity cut-points for the estimation of diabetes risk in British South Asians, African-Caribbeans and Europeans.

**Methods:**

We studied a population-based cohort from London, UK (1356 Europeans, 842 South Asians, 335 African-Caribbeans) who were aged 40–69 years at baseline (1988–1991), when they underwent anthropometry, fasting and post-load (75 g oral glucose tolerance test) blood tests. Incident Type 2 diabetes was identified from primary care records, participant recall and/or follow-up biochemistry. Ethnicity-specific obesity cut-points in association with diabetes incidence were estimated using negative binomial regression.

**Results:**

Diabetes incidence rates (per 1000 person years) at a median follow-up of 19 years were 20.8 (95% CI: 18.4, 23.6) and 12.0 (8.3, 17.2) in South Asian men and women, 16.5 (12.7, 21.4) and 17.5 (13.0, 23.7) in African-Caribbean men and women, and 7.4 (6.3, 8.7), and 7.2 (5.3, 9.8) in European men and women. For incidence rates equivalent to those at a BMI of 30 kg/m^2^ in European men and women, age- and sex-adjusted cut-points were: South Asians, 25.2 (23.4, 26.6) kg/m^2^; and African-Caribbeans, 27.2 (25.2, 28.6) kg/m^2^. For South Asian and African-Caribbean men, respectively, waist circumference cut-points of 90.4 (85.0, 94.5) and 90.6 (85.0, 94.5) cm were equivalent to a value of 102 cm in European men. Waist circumference cut-points of 84.0 (74.0, 90.0) cm in South Asian women and 81.2 (71.4, 87.4) cm in African-Caribbean women were equivalent to a value of 88 cm in European women.

**Conclusions:**

In prospective analyses, British South Asians and African-Caribbeans had equivalent diabetes incidence rates at substantially lower obesity levels than the conventional European cut-points.

**What's new?**

- Ethnicity-appropriate obesity cut-points as predictors of diabetes risk are much debated.
- Few longitudinal studies have addressed this topic, and none in the UK.
- This study followed a group of over 2500 people from three ethnic backgrounds for 19 years and identified that BMI levels of 25 kg/m^2^ in South Asians and 27 kg/m^2^ in African Caribbeans posed equivalent risk of developing diabetes to BMI of 30 kg/m^2^ in Europeans. Waist circumference equivalents were also lower in South Asians and African Caribbeans.
- These findings highlight the potential importance of public health measures for the prevention of metabolic risk long before the current conventional cut-points for obesity are reached in South Asian and African-Caribbean populations.

## Introduction

The definition of obesity [body mass index (BMI) ≥ 30 kg/m^2^], a key risk factor for Type 2 diabetes, has been validated in largely white populations. Ethnicity-appropriate obesity cut-points are much debated [1,2]. In 2002, the World Health Organization (WHO) Expert Panel concluded that there were insufficient data available to specify BMI cut-points that would be applicable to Asians, and put forward pragmatic, rather than evidence-based BMI cut-points for Asians: underweight, < 18.5 kg/m^2^; increasing but acceptable risk, 18.5 to < 23 kg/m^2^; increased risk, 23 to < 27.5 kg/m^2^; and high risk, ≥ 27.5 kg/m^2^ (i.e. defining obesity), compared with thresholds of 18.5, 25.0 and 30.0 kg/m^2^ in white Europeans [3]. No such guidance has been published for people of black African descent.

For waist circumference, similarly based on mostly European data, the WHO recommends cut-points of 102 cm (men) and 88 cm (women) as representing substantially increased risk of metabolic complications, broadly equivalent to BMI ≥ 30 kg/m^2^ [2,4]. These cut-points are widely used in the USA [5]. The International Diabetes Federation also recommends pragmatic sex- and ethnicity-specific cut-points for waist circumference in its definition of central obesity for Asian populations, and suggests the use of European cut-points for sub-Saharan African populations until more specific data become available [6].

Although several studies have attempted to provide BMI cut-points for South Asians, only one such study has used longitudinal data in a population with European comparators; this study also included cut-points for people of African descent [7]. In addition, one study has used longitudinal data to provide waist circumference cut-points for South Asians living in Mauritius and Europeans living in Australia [8].

Our aim was to identify prospectively levels of BMI and waist circumference for South Asian and African-Caribbean men and women which were comparable, in terms of diabetes risk, with the conventional cut-point for obesity of 30 kg/m^2^ for BMI and the waist circumference cut-points recommended by the WHO for Europeans.

## Methods

**Figure 1:**
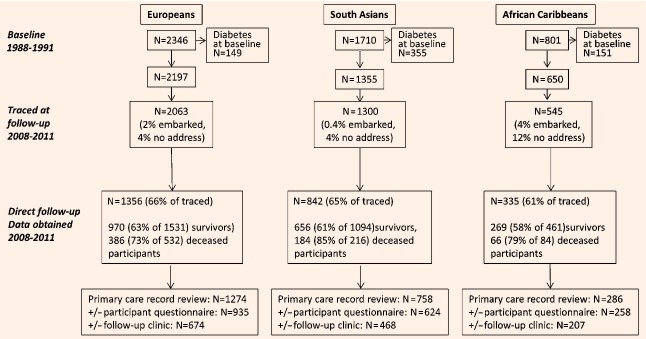
SABRE study flow chart (1988–2011).

Southall and Brent Revisited (SABRE) is a population-based cohort of Europeans, South Asians and African-Caribbeans from North and West London. Details of the cohort have been published previously [9]. People aged 40–69 years at baseline (1988–1991) were randomly selected from age- and sex-stratified primary care physician lists (*n* = 4063) and workplaces (*n* = 795) in the London boroughs of Southall and Brent (Fig. 1). The study was designed to investigate cardiometabolic risk in different ethnic groups, primarily in men.

All South Asians and African-Caribbeans were first-generation migrants. Ethnicity was confirmed based on parental origins. South Asians originated from the Indian subcontinent (the majority, 93% of 1710, were from India and Pakistan) and 64% (of 1710) were of Punjabi Sikh descent. African-Caribbeans originated from the Caribbean (91.5% of 801) or from West Africa. At baseline, participants underwent fasting blood tests, blood pressure measurements and anthropometry. Those whose diabetes status was unknown underwent oral glucose-tolerance testing.

### Baseline anthropometrics

All measurements were conducted using a standard protocol. Waist circumference was measured using a fibreglass tape with a spring balance set to a constant tension of 600 g. Height was measured using a stadiometer and volunteers were barefoot and wore a light hospital gown. Weight was measured using a Soehnle electronic scale. BMI was calculated as weight in kilograms divided by squared height in metres.

### Follow-up 2008–2011

During 2008–2011, surviving volunteers were invited to participate in a morbidity follow-up, including a health and lifestyle questionnaire, primary care medical record review and/or attendance at clinic at St Mary's Hospital, London. Clinic attendees fasted overnight and underwent measurements as at baseline, including oral glucose-tolerance testing.

### Follow-up response rates

At follow-up (2008–2011), of the 4202 participants without baseline diabetes, 3908 were traced to a UK address (94% of Europeans, 96% of South Asians and 84% of African-Caribbeans). We obtained data on follow-up diabetes status for 1054 and 302 European men and women (66% of those traced), 706 and 136 South Asian men and women (65%) and 189 and 146 African-Caribbean men and women (61%) (Fig. 1).

### Identifying baseline and incident diabetes

Physician diagnosis or WHO 1999 criteria [10] for fasting and oral glucose-tolerance testing blood glucose measurements defined baseline diabetes (for exclusion). Incident diabetes was identified from a positive report by one of the following sources of direct follow-up:

- primary care medical record review—recorded diagnosis of diabetes or prescription of anti-diabetic medications;
- participant questionnaire—recall of physician-diagnosed diabetes plus either year of diagnosis or receipt of named anti-diabetic medication;
- clinical follow-up at 20 years—fasting or oral glucose-tolerance testing plasma glucose results meeting WHO 1999 criteria (fasting plasma glucose ≥ 7 mmol/l or 2 h plasma glucose ≥ 11.1 mmol/l) [10].

Plasma glucose was measured using hexokinase/NADP methods (Abbott Diagnostics, Abbott Park, IL, USA).

Date of censoring was taken as date of diagnosis of diabetes, or last date of follow-up, or death (identified by the UK Office for National Statistics), if incident diabetes was not recorded.

### Statistical analyses

Unadjusted diabetes incidence rates were calculated by baseline BMI categories (using suggested WHO cut-points of < 23, 25, 27.5 and 30 kg/m^2^) and by baseline waist circumference in 10 cm categories [3].

We used negative binomial regression models with robust standard errors (due to evidence of overdispersion in Poisson models) to examine age- and sex-adjusted ethnicity-specific associations between baseline BMI or age-adjusted waist circumference and diabetes incidence rates. Inclusion of BMI and waist circumference as linear predictors of incident diabetes resulted in poorly fitted models, hence we examined the fit of models using the likelihood ratio test, adding a quadratic term and using restricted cubic splines with three, four and five knots. Models were selected according to the lowest Bayesian information criterion (BIC). For BMI, a negative binomial regression model, including BMI restricted cubic splines with three knots adjusted for age and sex, had a lower Bayesian information criterion than models containing BMI modelled as cubic splines with four or five knots or as a quadratic term. Similarly for waist circumference, Bayesian information criteria were lowest for both men and women in models in which waist circumference was modelled as sex-specific cubic splines with three knots.

Owing to non-linear associations with incident diabetes, age was included in the models in quartiles. *A priori*, we examined interactions between BMI or waist circumference variables and ethnicity, age and sex. We compared models with and without interaction terms using the likelihood ratio test. For our final models, we adopted the best-fit and most parsimonious models. We also examined analyses using 2.5 kg/m^2^ categories of BMI or 10 cm categories of waist circumference. We further examined the effects in multivariable models of adding conventional risk factors [baseline smoking category (never/ex/current), years of education, plasma triglyceride levels and systolic blood pressure]. In addition, we examined models that excluded deceased participants for whom the only form of follow-up was primary care record review. Finally, we examined models that excluded participants with prevalent coronary heart disease or stroke.

From the negative binomial regression models, we estimated predicted incidence rates and their 95% confidence intervals (95% CI) for European participants with a BMI of 30 kg/m^2^ or a waist circumference of 102 cm (men) and 88 cm (women) [4]. We identified the corresponding BMI values and 95% CI for South Asians and African-Caribbeans.

Because the widely accepted BMI obesity cut-point of 30 kg/m^2^ is applied to both men and women, we focused on findings for men and women combined. Analyses for waist circumference were conducted separately in men and women.

Statistical analyses were performed using Stata v. 12.

## Results

At baseline, mean age ± SD was 51.8 ± 7.0, range 40–72 years. South Asians were on average two years younger than Europeans and African-Caribbeans. On average, South Asians had lived in England for 23 years and African-Caribbeans for 30 years. Among men, mean BMI was similar in all three ethnic groups, although South Asian men were more centrally obese than European or African-Caribbean men. Among women, South Asians and African-Caribbeans had greater mean BMI, and were more centrally obese than Europeans. South Asian men and African-Caribbean women were the most insulin resistant (Table 1). Baseline characteristics for those without follow-up data (*n* = 1669) were generally similar or slightly less favourable than those with follow-up data (*n* = 2533) (Table S1).

**Table 1:** Baseline characteristics by ethnicity and sex (for those traced and followed up, and without diabetes at baseline): SABRE study, London

	Men			Women		
Means ± SD or geometric means (95% CI)	European	South Asian	African-Caribbean	European	South Asian	African-Caribbean
*N* (%)	1054 (78)	705 (84)	188 (56)	302 (22)	136 (16)	146 (44)
Age (years)	52.4 ± 7.1	50.6 ± 6.9	53.5 ± 6.0	52.6 ± 7.0	49.5 ± 6.4	52.2 ± 6.3
Age range	40–68	40–67	41–69	40–66	41–64	41–69
Years living in England (median, IQR)	–	23.5 (18.4, 26.9)	30.2 (28.4, 32.1)	–	20.4 (17.0, 23.8)	30.2 (28.1, 33.0)
BMI (kg/m^2^)	25.9 ± 3.7	25.5 ± 3.3	26.2 ± 3.2	25.8 ± 4.7	27.0 ± 4.3	29.5 ± 5.3
BMI categories (kg/m^2^)						
< 23	18%	21%	14%	31%	20%	8%
23 to < 25	24%	24%	24%	19%	17%	12%
25 to < 27.5	30%	32%	26%	23%	21%	12%
27.5 to < 30	16%	15%	26%	13%	18%	31%
30 to < 40	11%	7%	9%	13%	24%	32%
40+	0.2%	0.4%	0.5	1%	–	5%
Waist circumference (cm)	90.6 (90.0, 91.3)	91.7 (91.0, 92.4)	88.2 (86.9, 89.6)	78.5 (77.2, 79.8)	83.2 (81.5, 85.0)	87.2 (85.4, 89.2)
Waist categories (cm)						
< 80	12%	8%	19%	61%	38%	28%
80 to < 90	35%	34%	35%	23%	33%	33%
90 to < 100	33%	38%	31%	8%	21%	23%
100 +	19%	19%	14%	8%	7%	16%
Waist:hip ratio	0.94 ± 0.06	0.98 ± 0.06	0.93 ± 0.06	0.80 ± 0.8	0.85 ± 0.85	0.84 ± 0.07
Waist:height ratio	0.52 ± 0.06	0.54 ± 0.06	0.51 ± 0.05	0.49 ± 0.07	0.54 ± 0.07	0.54 ± 0.08
Fasting glucose (mmol/l)	5.4 (5.4, 5.4)	5.4 (5.4, 5.5)	5.5 (5.4, 5.6)	5.3 (5.2, 5.3)	5.0 (4.9, 5.1)	5.4 (5.3, 5.5)
Fasting insulin (uU/ml)	7.1 (6.9, 7.4)	9.8 (9.4,10.2)	7.6 (7.0, 8.3)	5.3 (5.0, 5.7)	6.9 (6.4, 7.5)	9.2 (8.4, 10.0)
HOMA 2 insulin resistance	0.82 (0.79, 0.85)	1.11 (1.07, 1.16)	0.88 (0.81, 0.96)	0.60 (0.56, 0.65)	0.78 (0.72, 0.84)	1.05 (0.96, 1.14)
Total cholesterol (mmol/l)	6.0 (5.9, 6.0)	5.9 (5.8, 6.0)	5.4 (5.3, 5.6)	6.0 (5.8, 6.1)	5.6 (5.4, 5.9)	5.4 (5.2, 5.6)
HDL cholesterol (mmol/l)	1.3 (1.2, 1.3)	1.2 (1.1, 1.2)	1.4 (1.3, 1.5)	1.6 (1.6, 1.6)	1.4 (1.4, 1.5)	1.6 (1.5, 1.7)
Triglycerides (mmol/l)	1.5 (1.4, 1.5)	1.7 (1.7, 1.8)	1.1 (1.0, 1.1)	1.3 (1.2, 1.3)	1.3 (1.2, 1.4)	1.0 (0.9, 1.1)
Systolic blood pressure (mm Hg)	122 ± 16	124 ± 17	127 ± 15	119 ± 17	123 ± 22	129 ± 16
Years of education	*N* = 1046	*N* = 679	*N* = 184	*N* = 301	*N* = 106	*N* = 145
	10.7 ± 2.6	12.5 ± 3.7	10.9 ± 2.8	10.7 ± 2.8	11.0 ± 3.6	10.8 ± 3.6
Smoking: current/ex/never (%)	33/39/28	16/10/74	24/20/56	29/24/47	2/1/97	12/8/81

The Homeostasis Model Asssessment (HOMA 2) Calculator was used to estimate insulin resistance: https://www.dtu.ox.ac.uk/homacalculator/.

During the median follow-up period of 19 years [interquartile range (IQR): 15, 20 years), diabetes developed in 578 participants (23%). These included 156 (15%) European men and 40 (13%) European women, 253 (36%) South Asian men and 29 (21%) South Asian women, and 56 African-Caribbean men (30%) and 44 (30%) African-Caribbean women. Median age (IQR) at onset of diabetes was 67 (60, 71) and 65 (62, 72) years in European men and women, 62 (57, 68) and 67 (57, 69) in South Asian men and women, and 68 (62, 72) and 63 (60, 67) in African-Caribbean men and women.

The majority of cases were identified from primary care medical record review (436; 75%) or clinic oral glucose-tolerance testing (15%). A small number (49) were identified from participant questionnaire alone and seven were identified from death certification alone. For 1516 participants with both a primary care record review and participant questionnaire, there was 97% agreement for diabetes diagnosis.

### Baseline BMI and incident diabetes

**Figure 2:**
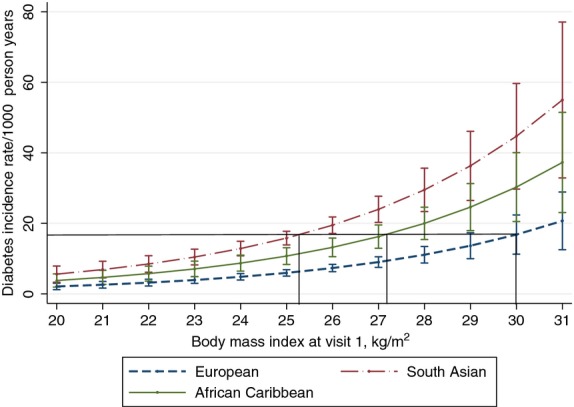
Diabetes incidence rates during 19 years of follow-up by baseline body mass index (men and women combined). Negative binomial regression model adjusted for age and sex.

Unadjusted diabetes incidence rates increased with increasing baseline BMI and, in men, were highest across all BMI categories in South Asians and lowest in Europeans. In women, unadjusted rates were highest in African-Caribbeans and lowest in Europeans (Table 2). Age- and sex-adjusted BMI cut-points of 25.2 (95% CI: 23.4, 26.6) kg/m^2^ in South Asians and 27.2 (25.2, 28.6) kg/m^2^ in African-Caribbeans were associated with diabetes incidence rates equivalent to that of 30 kg/m^2^ in Europeans (Fig. 2).

**Table 2:** Unadjusted diabetes incidence rates (95% CI) per 1000 person years by BMI and waist circumference category in men and women: SABRE study, London, 1988–1990 (baseline) to 2008–2011

	Men			Women		
	European	South Asian	African-Caribbean	European	South Asian	African-Caribbean
Number (%)	1054 (78)	706 (84)	188 (56)	302	136	146
Number of cases	156	253	56	40	29	44
Person years of follow-up	20 400	12 000	3400	5600	2400	2400
Overall incidence rates/1000 person years	7.4 (6.3, 8.7)	20.8 (18.4, 23.6)	16.5 (12.7, 21.4)	7.2 (5.3,9.8)	12.0 (8.3, 17.2)	17.5 (13.0, 23.7)
BMI (kg/m^2^)						
< 23	2.9 (1.6, 5.3)	9.5 (6.5, 13.9)	6.1 (2.0, 18.9)	3.4 (1.5, 7.7)	9.4 (3.9, 22.6)	4.8 (0.7, 33.8)
23 to < 25	4.0 (2.6, 6.2)	17.1 (12.9, 22.4)	10.4 (5.2, 20.7)	1.8 (0.5, 7.2)	2.2 (0.3, 16.0)	16.4 (6.8, 39.4)
25 to < 27.5	6.8 (5.1, 9.2)	21.3 (17.1, 26.5)	17.1 (10.6, 27.5)	6.6 (3.4, 12.7)	9.4 (3.9, 22.6)	6.3 (1.6, 25.3)
27.5 to < 30	10.3 (7.3, 14.6)	32.2 (24.4, 42.5)	18.8 (11.5, 30.6)	11.0 (5.2, 23.0)	14.3 (6.4, 31.9)	12.1 (6.3, 23.2)
30+	17.1 (12.4, 23.7)	39.2 (27.0, 56.7)	30.7 (15.3, 61.3)	23.8 (14.4,39.5)	22.5 (12.5, 40.6)	27.2 (17.4, 42.7)
Waist circumference (cm)						
< 80	2.5 (1.3, 5.8)	5.4 (2.4, 12.0)	3.1 (0.8, 12.2)	4.1 (2.4, 6.9)	9.0 (4.7, 17.4)	6.8 (2.8, 16.3)
80 to < 90	4.5 (3.2, 6.4)	14.8 (11.6, 18.9)	20.5 (13.9, 30.3)	7.1 (3.7,13.7)	8.8 (4.2, 18.4)	14.1 (7.8, 25.4)
90 to < 100	8.3 (6.4,10.8)	23.6 (19.5, 28.4)	14.2 (8.7, 23.1)	14.8 (6.6, 32.9)	19.5 (10.2,37.5)	23.4 (13.6, 40.3)
100+	15.2 (11.6, 19.8)	37.2 (29.5, 46.9)	30.5 (17.3, 53.8)	29.1 (16.1,52.5)	23.5 (8.8, 62.7)	40.6 (23.6, 69.9)

Further adjustment for years of education, smoking, triglycerides and systolic blood pressure barely altered the estimated ethnicity-specific cut-points for BMI [South Asians: 25.0 (22.5, 26.5) kg/m^2^; African-Caribbeans: 27.0 (25.0, 28.5) kg/m^2^]. Duration of residence in England was not significantly associated with diabetes incidence and was not included in multivariable models. An age- and sex-adjusted model that included BMI in 2.5 kg/m^2^ categories corresponded well in terms of the shape and position of regression curves with the final model, giving further support to the use of cubic splines with three knots in the final model (Fig. S1). Analyses that excluded deceased participants who had not completed questionnaires or attended our clinic prior to death suggested slightly higher cut-points [South Asians: 25.7 (23.5, 27.1) kg/m^2^; African-Caribbeans: 27.5 (25.3, 29.0) kg/m^2^].

### Baseline waist circumference and incident diabetes

**Figure 3:**
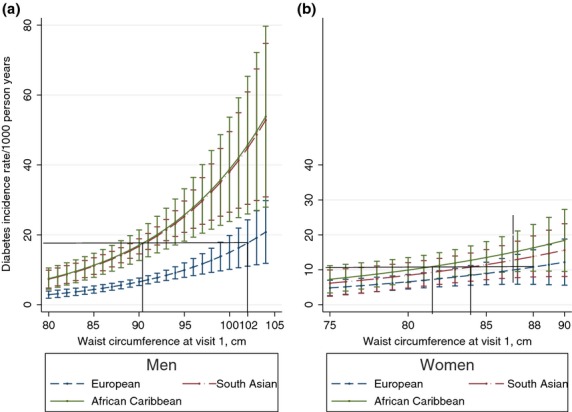
Diabetes incidence rates during 19 years of follow-up by baseline waist circumference, negative binomial regression model adjusted for age. (a) Men, (b) women.

Waist circumferences of 90.6 (85.0, 94.5) cm in South Asian men and 90.4 (85.0, 94.5) cm in African-Caribbean men were associated with similar age-adjusted incidence rates to European men with waist circumferences of 102 cm (Fig. 3a).

South Asian women with waist circumferences of 84.0 (74.0, 90.0) cm and African-Caribbean women with waist circumferences of 81.2 (71.4, 87.4) cm had equivalent diabetes incidence rates to European women with waist circumferences of 88 cm (Fig. 3b).

Adjustment for years of education, smoking status, triglycerides and systolic blood pressure lowered the waist circumference cut-points by ∼ 2 cm in ethnic minority men [South Asians: 88.4 (83.2, 92.2) cm, African-Caribbeans: 88.2 (83.0, 92.0) cm] and increased them by 1–2 cm in women [African-Caribbeans: 82.4 (70.2, 88.8) cm; South Asians: 85.6 (73.4, 92.0) cm]. Analyses that excluded deceased participants who had not completed questionnaires or attended our clinic prior to death did not alter the waist circumference cut-points.

Finally, exclusion of those with coronary heart disease or stroke at baseline did not alter the findings for either BMI or waist circumference.

## Discussion

In prospective analyses, we show striking ethnic differences in diabetes incidence at each level of BMI and waist circumference. Our findings suggest that in terms of diabetes incidence, mid-life BMI values of 25.2 kg/m^2^ in South Asians and 27.2 kg/m^2^ in African-Caribbeans were equivalent to the conventional cut-point of 30 kg/m^2^ in Europeans. For central obesity, South Asian and African-Caribbean men had almost identical and strong associations between increasing waist circumference and diabetes incidence, such that men with waist circumferences of 90.4–90.6 cm had equivalent rates to European men with waist circumferences of 102 cm. Waist circumferences of 84 and 81 cm, respectively, in South Asian and African-Caribbean women were associated with diabetes incidence rates equivalent to those of European women with waist circumferences of 88 cm.

Our findings for BMI suggest somewhat higher cut-points than a recent longitudinal community-based study in Canada, which reported multivariable-adjusted diabetes incidence rates in South Asians and Africans with BMI values of 24 and 26 kg/m^2^ equivalent to rates in Europeans with BMIs of 30 kg/m^2^ (men and women combined) [7]. Nonetheless, both that study and ours suggest greater metabolic risk at lower BMI cut-points among the two ethnic groups than among comparator populations of European origin. In our study, further adjustment for conventional risk factors, including years of education and smoking, or exclusion of those who died and for whom follow-up was limited to medical record review or death certificate, altered the estimates very little, and the ethnicity-specific cut-points remained substantially below the equivalent European cut-points for both BMI and waist circumference. Taken together, these longitudinal studies suggest robust findings that may be generalizable across similar migrant populations.

Several other groups have attempted to redefine obesity cut-points for people of Asian origin, but most were cross-sectional in design [11–21] and not all reported diabetes as an outcome [11,12].

Our findings raise the issue that obesity cut-off points lower than the conventional values may be warranted in immigrant South Asians and African-Caribbeans compared with the European white population in the UK, to pose equivalent risk for Type 2 diabetes. Inasmuch as the purpose of an anthropometric (e.g. BMI) cut-off point is to identify the proportion of people with a high risk of important health conditions in a population, it has the potential to inform public health action points, such as health promotion and prevention programmes. However, lowering the cut-off values in ethnic groups will have the obvious effect of increasing the prevalence of obesity among that population. At the individual level, the label of being obese would be assigned at lower BMI thresholds, with unknown consequences. Currently, there is lack of evidence on whether minority ethnic groups would respond to health behaviour interventions in a similar way to Europeans even at conventional obesity cut-offs, and nothing is known about the effectiveness of interventions if BMI thresholds to define obesity were lowered. However, one target could be to promote greater physical activity among ethnic minority groups, who have well-documented evidence for being less physically active than Europeans [22,23]. Encouragingly, there is evidence that targeted lifestyle intervention among people with impaired glucose tolerance is effective in preventing or delaying the onset of diabetes among South Asians in India [24]. We need to fill the research gap to identify if such lifestyle interventions could also work among minority ethnic group populations with obesity defined using lower cut-points. Taken collectively, these issues argue for public health action points for metabolic risk at lower BMI (and waist circumference) cut-points among the South Asian and African-Caribbean ethnic groups, but it would be important to not use these cut points to classify or label people as obese, which could incur unintended consequences.

### Strengths and weaknesses

SABRE is a longitudinal study with participants from three ethnic groups drawn from the same West and North-West London population. Study follow-up is lengthy (19 years) from middle age, with high numbers of diabetes cases in men. We objectively measured, rather than self-assessed, anthropometric data, and diabetes incidence was comprehensively assessed at the individual level via primary care record review, self-report, medication use and direct testing. Our analyses were comprehensive and the findings were robust to a range of sensitivity analyses. This is the first longitudinal study in the UK to report ethnicity- and sex-specific associations between mid-life obesity measures and incident diabetes and the findings are remarkably similar to the only other longitudinal study conducted in migrant populations. The inclusion of thresholds for waist circumference is novel.

We acknowledge that we have small numbers of women due to the initial study design and we would urge caution in considering our cut-points for women. Loss to follow-up is usual in cohort studies and follow-up data are missing for approximately one-third of participants in all ethnic groups, however, there were few significant differences in baseline characteristics of those with and without follow-up data and we would not anticipate differences in the associations between incident diabetes and baseline obesity measures in those lost to follow-up. Another limitation is that we have anthropometric data from only a single measurement made in mid-life.

We appreciate that BMI is not a good indicator of central adiposity and may not be the best indicator of diabetes risk across ethnic groups. However, our decision to include BMI in our analyses was pragmatic, given that it is frequently measured and understood, subject to less measurement error than waist and other circumferences and is the subject of numerous national and international guidelines and definitions of obesity.

South Asian and African-Caribbean study participants were all first generation migrants and these findings may not necessarily be applicable to future generations, or to South Asians and African-Caribbeans living in their home countries or countries other than the UK, or to those of mixed ethnicity. Our findings of the lower obesity cut-points in South Asians and African-Caribbeans compared with Europeans apply only to the outcome of type 2 diabetes, and may not apply to other endpoints such as cardiovascular disease or mortality. The rationale to choose diabetes as the outcome for these analyses is based on its progressive and strong associations with adiposity, and its rising prevalence and public health burden globally [26].

In conclusion, British South Asians and African-Caribbeans between the ages of 40 and 70 had increased risk of developing diabetes during 19 years of follow-up compared with Europeans of the same BMI or waist circumference. Our prospective findings suggest that BMI cut-off points of 25 kg/m^2^ in South Asians and 27 kg/m^2^ in African-Caribbeans pose equivalent risk for future diabetes as with BMI of 30 kg/m^2^ among Europeans. Similarly, lower thresholds of waist circumference among both ethnic minority groups pose equivalent diabetes risk to the conventional European cut-points. These findings suggest the potential importance of public health measures for the prevention of metabolic risk long before the current conventional cut-points for obesity are reached in South Asian and African-Caribbean populations.

## Supporting Information

**Figure S1**. BMI categories (men and women combined by ethnicity) and predicted probabilities (95% CI) of incident diabetes (adjusted for age and sex). **Table S1**. Baseline characteristics by ethnicity and sex in those included and those lost to follow-up and without baseline diabetes.

## References

[1] Wen CP, David ChengTY, Tsai SP, Chan HT, Hsu HL, Hsu CC (2009). Are Asians at greater mortality risks for being overweight than Caucasians? Redefining obesity for Asians. Public Health Nutr.

[2] Pan WH, Yeh WT (2008). How to define obesity? Evidence-based multiple action points for public awareness, screening, and treatment: an extension of Asian-Pacific recommendations. Asia Pac J Clin Nutr.

[3] World Health Organization (2004). Appropriate body-mass index for Asian populations and its implications for policy and intervention strategies. Lancet.

[4] World Health Organization (2008). http://www.who.int/nutrition/publications/obesity/WHO_report_waistcircumference_and_waisthip_ratio/en/.

[5] Expert Panel on Detection (2001). Evaluation, and Treatment of High Blood Cholesterol in Adults. Executive Summary of the Third Report of The National Cholesterol Education Program (NCEP) Expert Panel on Detection, Evaluation, and Treatment of High Blood Cholesterol in Adults (Adult Treatment Panel III). JAMA.

[6] International Diabetes Federation (2006). http://www.idf.org/webdata/docs/MetSyndrome_FINAL.pdf.

[7] Chiu M, Austin PC, Manuel DG, Shah BR, Tu JV (2011). Deriving ethnic-specific BMI cutoff points for assessing diabetes risk. Diabetes Care.

[8] Cameron AJ, Sicree RA, Zimmet PZ, Alberti KG, Tonkin AM, Balkau B (2010). Cut-points for waist circumference in Europids and South Asians. Obesity (Silver Spring).

[9] Tillin T, Forouhi NG, McKeigue PM, Chaturvedi N (2012). Southall And Brent REvisited: cohort profile of SABRE, a UK population-based comparison of cardiovascular disease and diabetes in people of European, Indian Asian and African Caribbean origins. Int J Epidemiol.

[10] World Health Organization (1999). Definition, Diagnosis and Classification of Diabetes Mellitus and its Complications. Part 1: Diagnosis and classification of diabetes mellitus.

[11] Jafar TH, Chaturvedi N, Pappas G (2006). Prevalence of overweight and obesity and their association with hypertension and diabetes mellitus in an Indo-Asian population. CMAJ.

[12] Zhou BF (2002). Predictive values of body mass index and waist circumference for risk factors of certain related diseases in Chinese adults – study on optimal cut-off points of body mass index and waist circumference in Chinese adults. Biomed Environ Sci.

[13] Razak F, Anand S, Vuksan V, Davis B, Jacobs R, Teo KK (2005). Ethnic differences in the relationships between obesity and glucose-metabolic abnormalities: a cross-sectional population-based study. Int J Obes (Lond).

[14] Gray LJ, Yates T, Davies MJ, Brady E, Webb DR, Sattar N (2011). Defining obesity cut-off points for migrant South Asians. PLoS ONE.

[15] Ko GT, Chan JC, Cockram CS, Woo J (1999). Prediction of hypertension, diabetes, dyslipidaemia or albuminuria using simple anthropometric indexes in Hong Kong Chinese. Int J Obes Relat Metab Disord.

[16] Oh SW, Shin SA, Yun YH, Yoo T, Huh BY (2004). Cut-off point of BMI and obesity-related comorbidities and mortality in middle-aged Koreans. Obes Res.

[17] Deurenberg-Yap M, Deurenberg P (2003). Is a re-evaluation of WHO body mass index cut-off values needed? The case of Asians in Singapore. Nutr Rev.

[18] Li G, Chen X, Jang Y, Wang J, Xing X, Yang W (2002). Obesity, coronary heart disease risk factors and diabetes in Chinese: an approach to the criteria of obesity in the Chinese population. Obes Rev.

[19] Tseng CH (2006). Body mass index and waist circumference as determinants of coronary artery disease in Taiwanese adults with type 2 diabetes mellitus. Int J Obes (Lond).

[20] Wildman RP, Gu D, Reynolds K, Duan X, He J (2004). Appropriate body mass index and waist circumference cutoffs for categorization of overweight and central adiposity among Chinese adults. Am J Clin Nutr.

[21] Mohan V, Deepa M, Farooq S, Narayan KM, Datta M, Deepa R (2007). Anthropometric cut points for identification of cardiometabolic risk factors in an urban Asian Indian population. Metabolism.

[22] Fischbacher CM, Hunt S, Alexander L (2004). How physically active are South Asians in the United Kingdom? A literature review. J Public Health (Oxf).

[23] Williams ED, Stamatakis E, Chandola T, Hamer M (2011). Assessment of physical activity levels in South Asians in the UK: findings from the Health Survey for England. J Epidemiol Community Health.

[24] Ramachandran A, Snehalatha C, Mary S, Mukesh B, Bhaskar AD, Vijay V (2006). The Indian Diabetes Prevention Programme shows that lifestyle modification and metformin prevent type 2 diabetes in Asian Indian subjects with impaired glucose tolerance (IDPP-1). Diabetologia.

[25] Tillin T, Hughes AD, Godsland IF, Whincup P, Forouhi NG, Welsh P (2013). Insulin resistance and truncal obesity as important determinants of the greater incidence of diabetes in Indian Asians and African Caribbeans compared with Europeans: the Southall And Brent REvisited (SABRE) cohort. Diabetes Care.

[26] Whiting DR, Guariguata L, Weil C, Shaw J (2011). IDF diabetes atlas: global estimates of the prevalence of diabetes for 2011 and 2030. Diabetes Res Clin Pract.

