# Supplementary material for: Ethnicity-specific obesity cut-points in the development of Type 2 diabetes – a prospective study including three ethnic groups in the United Kingdom
Source: Diabet Med. 2014 Oct 1;32(2):226–34. doi: 10.1111/dme.12576 (PMC4441277; doi:10.1111/dme.12576)
Supplement: Supplementary file 1 [file dme0032-0226-sd1.docx]

Table S1 Baseline characteristics by ethnicity and sex in those included and those lost to follow-up and without baseline diabetes.

| European | Men |  |  | Women |  |  |
| --- | --- | --- | --- | --- | --- | --- |
| Means ± SD or geometric means (95% CI) | Included | Lost to follow-up | P value | Included | Lost to follow-up | P value |
| Number | 1054 (78%) | 607 |  | 302 (22%) | 233 |  |
| Age: years | 52.4 ± 7.1 | 53.0 ± 7.2 | 0.13 | 52.6 ± 7.0 | 53.5 ± 6.3 | 0.11 |
| range | 40–68 | 40–67 |  | 40–66 | 41–66 |  |
| BMI, kg/m^2^ | 25.9 ± 3.7 | 26.3 ± 3.9 | 0.045 | 25.8 ± 4.7 | 26.0 ± 4.4 | 0.5 |
| Waist circumference, cm | 90.6 (90.0, 91.3) | 91.5 (90.7,92.4) | 0.08 | 78.5 (77.2, 79.8) | 78.9 (77.4, 80.4) | 0.7 |
| Fasting glucose, mmol/l | 5.4 (5.4, 5.4) | 5.4 (5.3, 5.4) | 0.7 | 5.3 (5.2, 5.3) | 5.2 (5.2, 5.3) | 0.2 |
| Fasting insulin, uU/ml | 7.1 (6.9, 7.4) | 7.0 (6.7, 7.3) | 0.5 | 5.3 (5.0, 5.7) | 5.6 (5.1, 6.0) | 0.2 |
| HOMA 2 insulin resistance | 0.82 (0.79, 0.85) | 0.80 (0.76, 0.83) | 0.4 | 0.60 (0.56, 0.65) | 0.63 (0.58, 0.68) | 0.4 |
| Triglycerides, mmol/l | 1.5 (1.4, 1.5) | 1.5 (1.4, 1.6) | 0.6 | 1.3 (1.2, 1.3) | 1.3 (1.2, 1.3) | 0.7 |
| SBP, mm Hg | 122 ± 16 | 124 ± 17 | 0.018 | 119 ± 17 | 120 ± 15 | 0.7 |
| Years of education | N = 1046  10.7 ± 2.6 | 10.5 ± 2.5 | 0.050 | N = 301  10.7 ± 2.8 | 9.9 ± 2.5 | 0.002 |
| Smoking: current/ex/never % | 33/39/28 | 35/39/25% | 0.4 | 29/24/47 | 31/21/48 | 0.8 |
| South Asian | Men |  |  | Women |  |  |
| Means ± SD or geometric means (95% CI) | Included | Lost to follow-up | P value | Included | Lost to follow-up | P value |
| Number | 705 (84%) | 405 |  | 136 (16%) | 108 |  |
| Age: years | 50.6 ± 6.9 | 50.3 ± 6.9 | 0.63 | 49.5 ± 6.4 | 50.8 ± 7.0 | 0.14 |
| range | 40–67 | 40–67 |  | 41–64 | 41–66 |  |
| BMI, kg/m^2^ | 25.5 ± 3.3 | 25.9 ± 3.3 | 0.062 | 27.0 ± 4.3 | 27.1 ± 4.5 | 0.8 |
| Waist circumference, cm | 91.7 (91.0, 92.4) | 92.4 (91.4,93.3) | 0.3 | 83.2 (81.5, 85.0) | 84.5 (82.4, 86.8) | 0.3 |
| Fasting glucose, mmol/l | 5.4 (5.4, 5.5) | 5.4 (5.4, 5.5) | 0.7 | 5.0 (4.9, 5.1) | 5.0 (5.0, 5.1) | 0.3 |
| Fasting insulin, uU/ml | 9.8 (9.4,10.2) | 9.8 (9.3, 10.4) | 0.9 | 6.9 (6.4, 7.5) | 8.0 (7.2, 9.0) | 0.03 |
| HOMA 2 insulin resistance | 1.11 (1.07, 1.16) | 1.12 (1.05, 1.19) | 0.9 | 0.78 (0.72, 0.84) | 0.90 (0.81, 1.01) | 0.03 |
| Triglycerides, mmol/l | 1.5 (1.4, 1.5) | 1.7 (1.6, 1.8) | 0.9 | 1.3 (1.2, 1.4) | 1.4 (1.3, 1.5) | 0.5 |
| SBP, mm Hg | 122 ± 16 | 124 ± 17 | 0.7 | 123 ± 22 | 123 ± 17 | 1.0 |
| Years of education | N = 1046  12.5 ± 3.6 | 12.2 ± 3.6 | 0.12 | N = 106  11.0 ± 3.6 | 9.4 ± 3.3 | 0.003 |
| Smoking: current/ex/never, % | 33/39/28 | 15/9/75 | 0.8 | 2/1/97 | 2/0/98 | 0.7 |
| African Caribbean | Men |  |  | Women |  |  |
|  | Included | Lost to follow-up | P value | Included | Lost to follow-up | P value |
| Number | 188 (56%) | 182 |  | 146 (44%) | 133 |  |
| Age: years | 53.5 ± 6.0 | 53.5 ± 5.7 | 0.97 | 52.2 ± 6.3 | 53.1 ± 5.7 | 0.22 |
| range | 41–69 | 40–69 |  | 41–69 | 41–64 |  |
| BMI, kg/m2 | 26.2 ± 3.2 | 26.1 ± 3.2 | 0.6 | 29.5 ± 5.3 | 29.3 ± 4.4 | 0.7 |
| Waist circumference, cm | 88.2 (86.9, 89.6) | 88.5 (87.1,89.9) | 0.9 | 87.2 (85.4, 89.2) | 87.5 (85.6, 89.4) | 0.9 |
| Fasting glucose, mmol/l | 5.5 (5.4, 5.6) | 5.5 (5.4,5.6) | 0.9 | 5.0 (4.9, 5.1) | 5.5 (5.4, 5.6) | 0.5 |
| Fasting insulin, uU/ml | 7.6 (7.0, 8.3) | 7.6 (7.0, 8.3) | 0.8 | 6.9 (6.4, 7.5) | 7.8 (6.9, 8.8) | 0.035 |
| HOMA 2 insulin resistance | 0.88 (0.81, 0.96) | 0.87 (0.80, 0.95) | 0.9 | 0.78 (0.72, 0.84) | 0.92 (0.83, 1.02) | 0.060 |
| Triglycerides, mmol/l | 1.1 (1.0, 1.1) | 1.1 (1.0, 1.2) | 0.2 | 1.3 (1.2, 1.4) | 1.0 (1.0, 1.1) | 0.5 |
| SBP, mm Hg | 127 ± 15 | 128 ± 19 | 0.4 | 123 ± 22 | 130 ± 16 | 0.7 |
| Years of education | N = 184  10.9 ± 2.8 | 10.4 ± 3.0 | 0.10 | N = 106  10.8 ± 3.1 | 10.2 ± 2.8 | 0.059 |
| Smoking: current/ex/never, % | 24/20/56 | 35/18/47 | 0.067 | 2/1/97 | 5/8/87 | 0.10 |

Figure S1 BMI categories (men and women combined by ethnicity) and predicted probabilities (95% CI) of incident diabetes (adjusted for age and sex)
